# Three-Year Mortality of Older Hospitalized Patients with Osteosarcopenia: Data from the OsteoSys Study

**DOI:** 10.3390/nu16091328

**Published:** 2024-04-28

**Authors:** Maryam Pourhassan, Bjoern Buehring, Ulrik Stervbo, Sven Rahmann, Felix Mölder, Sebastian Rütten, Nina Rosa Neuendorff, Timm Henning Westhoff, Nina Babel, Rainer Wirth

**Affiliations:** 1 Department of Geriatric Medicine, Marien Hospital Herne, Ruhr-University Bochum, Hölkeskampring 40D, 44625 Herne, Germany; ninarosa.neuendorff@elisabethgruppe.de (N.R.N.); rainer.wirth@elisabethgruppe.de (R.W.); 2 Rheumazentrum Ruhrgebiet, Ruhr-University Bochum, 44649 Herne, Germany; bjoern.buehring@rub.de; 3 Bergisches Rheuma-Zentrum Wuppertal, 42105 Wuppertal, Germany; 4 Center for Translational Medicine and Immune Diagnostics Laboratory, Medical Department I, Marien Hospital Herne, Ruhr-University Bochum, 44625 Herne, Germany; ulrik.stervbo@elisabethgruppe.de (U.S.); nina.babel@elisabethgruppe.de (N.B.); 5 Algorithmic Bioinformatics, Center for Bioinformatics Saar, Saarland University, Saarland Informatics Campus, 66123 Saarbrücken, Germany; rahmann@cs.uni-saarland.de; 6 Algorithms for Reproducible Bioinformatics, Genome Informatics, Institute of Human Genetics, University Hospital Essen, University of Duisburg-Essen, 45147 Essen, Germany; felix.moelder@uni-due.de; 7 Institute of Pathology, University Hospital Essen, University of Duisburg-Essen, 45147 Essen, Germany; 8 Center for Orthopedics and Trauma Surgery, St. Anna Hospital, St. Elisabeth Gruppe, 44649 Herne, Germany; sebastian.ruetten@elisabethgruppe.de; 9 Charité-Universitätsmedizin Berlin, Corporate Member of Freie Universität Berlin, Humboldt-Universität zu Berlin, and Berlin Institute of Health, 10117 Berlin, Germany

**Keywords:** sarcopenia, osteopenia, osteoporosis, osteosarcopenia, bone mineral density, mortality, unplanned hospitalization

## Abstract

Osteosarcopenia, the concurrent presence of sarcopenia and osteopenia/osteoporosis, poses a significant health risk to older adults, yet its impact on clinical outcomes is not fully understood. The aim of this prospective, longitudinal multicentre study was to examine the impact of osteosarcopenia on 3-year mortality and unplanned hospitalizations among 572 older hospitalized patients (mean age 75.1 ± 10.8 years, 78% female). Sarcopenia and low bone mineral density (BMD) were evaluated using Dual Energy X-ray Absorptiometry and the European Working Group on Sarcopenia in Older People (EWGSOP2) and WHO criteria, respectively. Among participants, 76% had low BMD, 9% were sarcopenic, and 8% had osteosarcopenia. Individuals with osteosarcopenia experienced a significantly higher rate of mortality (46%, *p* < 001) and unplanned hospitalization (86%, *p* < 001) compared to those without this condition. Moreover, “healthy” subjects—those without sarcopenia or low BMD—showed markedly lower 3-year mortality (9%, *p* < 001) and less unplanned hospitalization (53%, *p* < 001). The presence of osteosarcopenia (*p* = 0.009) increased the 3-year mortality risk by 30% over sarcopenia alone and by 8% over low BMD alone, underscoring the severe health implications of concurrent muscle and bone deterioration. This study highlights the substantial impact of osteosarcopenia on mortality among older adults, emphasizing the need for targeted diagnostic and therapeutic strategies.

## 1. Introduction

Osteoporosis and sarcopenia are common among older adults, with significant implications for health and quality of life. Osteoporosis, characterized by a reduction in bone mineral density (BMD), has been closely linked to an increased risk of osteoporotic fractures [1]. The World Health Organization (WHO) has established criteria for diagnosing osteoporosis, defining it as a BMD T-score that is 2.5 standard deviations (SDs) or more below the average of a young adult reference population of the same sex. Osteopenia, a less severe condition, however, indicating the imminent development of osteoporosis, is defined by a T-score between −1.0 and −2.5 SD [2].

On the other hand, sarcopenia is recognized as a systematic muscle disorder marked by diminished muscle strength, mass, and functionality [3], which contributes to an elevated risk of falls, disability, morbidity, hospitalization, and mortality [4]. While the criteria for diagnosing osteoporosis have been widely agreed upon since 1994, sarcopenia has faced challenges in achieving a universally accepted definition and diagnostic approach, leading to significant variations in prevalence estimates across studies. This variability can be as pronounced as a 40% difference when applying different diagnostic criteria to the same population [5,6]. Nevertheless, the updated guidelines from the European Working Group on Sarcopenia in Older People (EWGSOP2) have gained broad acceptance [7] and were accordingly applied in the current study.

The dynamic interplay between muscle and bone becomes increasingly critical in understanding the concurrent presentation of osteoporosis/osteopenia and sarcopenia, particularly in the geriatric population. This relationship underscores a biomechanical and biochemical interaction that significantly impacts mobility, stability, and overall health in older adults [8]. The interaction between muscle and bone is framed by the mechanostat theory, which suggests that bone tissue adapts to the mechanical loads imposed by muscle contractions and gravity, thereby preserving skeletal integrity and functionality [9]. Consequently, a decrease in muscle mass and strength, as seen in sarcopenia, directly compromises bone density and structure, elevating the risk for osteoporosis and related fractures [7].

Moreover, beyond the physical interactions, previous research underscores the importance of the biochemical interplay between muscle and bone, mediated by signalling molecules such as myokines from muscle fibres and osteokines from bone cells [10,11]. These molecules play a critical role in modulating bone formation, resorption, and muscle metabolism, thus highlighting a biochemical dialogue that contributes to the co-regulation of bone and muscle health. For instance, osteocalcin, produced by osteoblasts, has been shown to influence muscle energy metabolism, while myostatin, a myokine, can affect bone remodelling [10,11]. However, it remains unclear whether muscle atrophy directly leads to a decrease in bone mass or if the reduction in bone mass partly generates muscle atrophy. The bidirectional nature of this relationship suggests a complex feedback loop where the deterioration of one component could potentially exacerbate the decline of the other, further complicating the understanding of causality between muscle loss and bone demineralization. Enhancing our understanding of physiological muscle-bone interactions may lead to the development of novel interventions aiming at mitigating the morbidity associated with both conditions, thereby improving the well-being of older persons.

In this regard, osteoporosis/osteopenia and sarcopenia are now increasingly recognized under the combined term ‘osteosarcopenia’, signifying a condition where low bone density and muscle atrophy with functional decline coexist. The diagnostic criteria for osteosarcopenia, however, remain a subject of debate. Variability exists in the literature, with some definitions encompassing osteopenia and sarcopenia and others specifying the co-occurrence of osteoporosis and sarcopenia. This inconsistency highlights the need for a unified diagnostic framework to accurately identify and address osteosarcopenia. However, osteosarcopenia is associated with a broad spectrum of health complications, including but not limited to chronic diseases [12], endocrine dysfunctions [13], increased frailty [12], nutritional deficiencies [14], and diminished physical functions [15]. Osteosarcopenia is a unique syndrome that significantly deteriorates health outcomes, leading to increased dependency and a marked decline in the quality of life, particularly among older and frail populations [16]. Dietary factors play a crucial role in the prevention and management of both osteoporosis and sarcopenia, impacting the risk and severity of osteosarcopenia. Previous studies have underscored the pivotal roles of dietary calcium, protein, and vitamin D in managing osteosarcopenia [17,18,19]. The adequate intake of these nutrients supports bone health and muscle function, which are essential in treating osteosarcopenia [19].

Osteosarcopenia has been shown to significantly increase the risks of falls, fractures, and mortality in older adults [20,21,22,23]. This was highlighted in a recent longitudinal study involving 1044 community-dwelling women aged 75, known as the OPRA Cohort, which observed participants over a decade to assess the prevalence of osteosarcopenia and its comparative impact on the risks of fractures and mortality against the risk of low bone mass alone. The study revealed that individuals with osteosarcopenia faced a markedly higher risk of hip and major osteoporotic fractures, as well as an increased mortality rate, when compared to those with only low bone mass. Notably, in that study, the prevalence of confirmed osteosarcopenia rose from 3.0% at age 75 to 9.2% by age 85, underscoring its significant prevalence and relevance for health outcomes in older individuals [24]. Despite existing studies addressing the link between osteosarcopenia and its adverse outcomes, the extent to which the simultaneous presence of osteoporosis/osteopenia and sarcopenia increases these risks, beyond the individual impact of each condition, has not been clearly defined. This current study seeks to examine the effects of sarcopenia and low BMD on clinical outcomes, particularly focusing on mortality and the incidence of unplanned hospitalizations. Moreover, this study aims to determine if osteosarcopenia poses an increased risk for these adverse outcomes in comparison to sarcopenia or low BMD alone in older hospitalized patients.

## 2. Subjects and Methods

The OsteoSys study, conducted across three hospitals in Herne, Germany (Center for Orthopedics and Trauma Surgery, St. Anna Hospital (STA); Rheumatology Center Herne (RZR); and Marien Hospital Herne (MHH)), from February 2017 to October 2019 is a prospective observational multicentre investigation into the effects of osteoporosis in relation to chronic inflammation and cardiovascular complications among older hospitalized patients. A detailed description of the study population and methods has been reported in more detail elsewhere [25]. Briefly, consecutively hospitalized participants with known or suspected osteoporosis and who provided written informed consent were included. Data of the study were managed using the REDCap electronic data capture tool [26] hosted at Marien Hospital Herne. This secure, web-based software provided an efficient and reliable means of collecting and storing participant data, ensuring accuracy and consistency throughout the study.

In the initial design of our study, the primary focus was on the clinical assessment of sarcopenia and BMD in hospitalized patients, utilizing the EWGSOP2 and WHO criteria. Although nutritional status is recognized as a pivotal factor in the progression and management of osteosarcopenia, detailed dietary analyses, including the energy value of a diet, protein intake, calcium and vitamin D intake, and dairy product consumption, were not included in this study. A geriatric assessment was conducted in the initial days following hospital admission. The FRAIL scale was utilized to identify frailty, categorizing participants as not frail (score of 0), prefrail (scores of 1–2), and frail (scores of 3–5) [27]. The SARC-F questionnaire was employed to assess the risk of sarcopenia, with scores up to 10; individuals with scorings of ≥4 were considered to have probable sarcopenia [28]. A short physical performance battery (SPPB) was used to measure physical performance with scores of 8 or below, denoting impaired physical performance [29]. Measurements of handgrip strength were taken using a Jamar-type dynamometer (Lafayette Instrument Company, Lafayette, LA, USA, inMHH and Leonardo Mechanograph GF, Novotec Medical GmbH, Pforzheim, Germany, in RZR and STA), with tests performed three times on the dominant or unaffected side to ensure accuracy, noting the highest score achieved. Additionally, all participants were queried about any previous osteoporosis diagnoses.

Sarcopenia evaluation was aligned with the EWGSOP2’s updated criteria [7], incorporating the assessment of handgrip strength, appendicular muscle mass (ASM), and the SPPB. For handgrip strength, thresholds were set below 27 kg for men and 16 kg for women to indicate weakness. ASM was quantified using Dual Energy X-ray Absorptiometry (DXA), with values under 20 kg for men and 15 kg for women signalling low muscle mass. The SPPB, assessing lower extremity function, utilized a cut-off of ≤8 points to denote reduced physical performance for both sexes. According to the EWGSOP2 consensus, sarcopenia was defined as probable when low muscle strength was detected. A sarcopenia diagnosis was confirmed by the presence of low muscle strength and low ASM. Sarcopenia was considered severe when low handgrip strength, low ASM, and low SPPB were all detected. Conversely, individuals meeting none of these criteria were considered non-sarcopenic.

DXA scans using Lunar Prodigy Advance (GE Medical Systems, Madison, WI, USA) measured BMD and body compositions across all three centres. Patients, positioned supinely, had BMD assessed at the lumbar spine, total femur, and femoral neck in one session. Quality assurance was carried out on a regular basis, and CV% was less than 1 for all measurement sites. The diagnosis followed German and international guidelines [30,31], using the lowest T-score among the measured sites, including the lumbar spine, where the average of lumbar segments L1 to L4 served as the lumbar measure. Vertebral bodies with hardware, degenerative changes, or fractures were excluded according to available guidelines. Osteoporosis and osteopenia were defined as per WHO criteria [2], with T-scores of ≤−2.5 and between −1.0 and −2.5 SD, respectively, indicating a low BMD at a T-score of <−1.0. The presence of both low BMD and sarcopenia confirmed osteosarcopenia.

### 2.1. Outcomes Data

Outcome data, including survival status and unplanned hospitalizations, were collected through telephone interviews with either the patients themselves, if capable, or their relatives. In cases where direct communication was not feasible, information was sought from the family doctor, hospital information system, and municipal registration office. Unplanned hospitalization was defined as any hospital admission that occurred unexpectedly and was not scheduled or planned in advance. This includes admissions due to acute medical conditions and the unexpected worsening of chronic illnesses. The initiation of the follow-up period was marked by the patient’s discharge date from the hospital. During the telephone interviews, detailed inquiries were made regarding mortality (including the date and probable cause of death) and unplanned hospitalizations, specifying the reasons for hospitalization, such as falls, fractures, heart attacks, coronary interventions, congestive heart failure, strokes, lung infections, and cancer.

### 2.2. Data Analysis

Statistical analyses were conducted utilizing SPSS software (SPSS Statistics for Windows, Version 29.0, IBM Corp, Armonk, NY, USA). To assess the normality of the distribution of continuous variables, the Shapiro–Wilk test was utilized. Based on the results of this test, for variables adhering to a normal distribution, means, and standard deviations (SDs) were calculated, while non-normally distributed data were summarized using median values and interquartile ranges (IQRs). Categorical data were represented as frequencies and percentages. The Pearson Chi-square test facilitated comparisons between groups for categorical variables. To identify the impact of risk factors such as sarcopenia, BMD, osteosarcopenia, nutritional status, gender, and age and BMI (independent variables) on mortality (dependent variable), binary logistic regression analysis was employed. A significance threshold was set at *p* < 0.05.

## 3. Results

### 3.1. Characteristics of Study Participants

In our previously published baseline analysis of the OsteoSys study involving 890 patients, we focused on the prevalence of sarcopenia and its overlap with low BMD in a subset of 572 patients (mean age of 75.1 ± 10.8 years, 78% females) after excluding those without whole-body DXA scans (*n* = 318), essential for sarcopenia diagnosis according to the EWGSOP2 definition.

The main baseline characteristics of study participants are summarized in Table 1. Briefly, using the frail simple score, 41% of participants were classified as frail, while 49% were regarded as prefrail, with the remaining 11% being nonfrail. According to SARC-F, 59% had probable sarcopenia. SPPB was measured in 302 patients in which almost half of the participants (49%) showed poor physical performance (SPPB < 8). Furthermore, 20% of the participants reported previously known osteoporosis. SPPB was missing in 270 patients due to acute disease. Frail simple scores and SARC-F questionnaires were not completed for 14 and 11 patients, respectively, due to logistical challenges encountered during data collection.

Moreover, nutritional assessment using MNA-SF in a subgroup of 273 patients from MHH indicated that 26% were malnourished. The major reasons for hospitalization included cardiovascular diseases, post-stroke care, pneumonia, urinary tract infections, osteoporosis, falls and fractures, osteoarthritis, and rheumatologic diseases. Out of 572 patients, 394 patients (69%) had normal handgrip strength and were classified as nonsarcopenic, and 178 patients (31%) had low handgrip strength and were classified as probable sarcopenic according to the criteria of EWGSOP2. Due to the presence of low ASM, sarcopenia was confirmed in 52 patients (29%, 52/178, or 9%, 52/572, of the total population), of which 25 patients had low SPPB and fulfilled the criteria for severe sarcopenia (of those with confirmed sarcopenia, SPPB values were missing for 19 patients). Out of 178 patients with probable sarcopenia, 126 patients had normal muscle mass and were classified as nonsarcopenic. Moreover, 76% (*n* = 435) exhibited low BMD, with osteopenia and osteoporosis present in 43% (*n* = 245) and 33% (*n* = 190) of patients, respectively. Notably, 8% (*n* = 47) of the participants were identified with osteosarcopenia, characterized by the co-presence of both low BMD and sarcopenia. Further detailed results of baseline data, including statistical analyses and comprehensive findings, are presented elsewhere [25].

### 3.2. Post-Discharge Mortality and Unplanned Hospitalization Outcomes

Post-discharge patient outcomes are detailed in Table 2. Out of the initial 572 baseline participants, follow-up data on mortality and unplanned hospitalization were available for 533 (132 patients from RZR, 128 patients from STA, and 273 patients from MHH) and 490 patients (124 patients from RZR, 125 patients from STA, and 241 patients from MHH), respectively. This discrepancy resulted in the exclusion of 39 participants (approximately 6.8%) for mortality analysis and 82 (approximately 14.3%) for hospitalization analysis due to the unavailability of their follow-up data. The median follow-up duration for the study was 4 years, with an interquartile range of 4 to 5 years, and the minimum follow-up period was 3 years. Given that the minimum follow-up encompassed all patient data, we opted to report the mortality rate at 3 years. Furthermore, the median time to death was 3 years, with an interquartile range of 1 to 4 years. Throughout the follow-up period, 30% of patients experienced mortality, with a 3-year mortality rate of 20% within the cohort. Unplanned hospitalization occurred in 60% of the subset, with 14% due to falls and 8% resulting from fractures.

### 3.3. Comparison of Outcome Data between Groups

In Table 3, the outcomes of 3-year mortality and unplanned hospitalization are compared between groups:

Sarcopenia vs. no sarcopenia: There was a significantly higher rate of 3-year mortality in the sarcopenia group, with 41% experiencing mortality, compared to 18% in the non-sarcopenic group (*p* < 0.001). Unplanned hospitalizations also occurred more frequently in sarcopenic patients in contrast to the non-sarcopenic group (*p* = 0.001). However, when examining the specific reasons for unplanned hospitalization, the difference between the groups was not statistically significant for falls or fractures.

Low BMD vs. normal BMD: The data reveal a substantial disparity in 3-year mortality rates between individuals with low versus normal BMD, with the former group exhibiting a mortality rate of 24%, significantly higher than the 9% observed in the latter (*p* < 0.001). Notably, hospitalization and unplanned hospitalizations due to falls were significantly more common in the low BMD group compared to the normal BMD group.

Osteosarcopenia vs. no osteosarcopenia: Our findings revealed that those with osteosarcopenia experienced a significantly higher mortality rate compared to the non-osteosarcopenic group (*p* < 0.001). A similar pattern was noted in unplanned hospitalization rates (*p* < 0.001).

In our analysis, individuals with normal bone and muscle status (*n* = 132), who had neither sarcopenia nor low BMD, exhibited a substantially lower 3-year mortality rate of 9% (12 out of 130) in contrast to the 46% mortality observed in patients with osteosarcopenia (*p* < 0.001). Furthermore, the incidence of unplanned hospitalizations in the “healthy” cohort was significantly less at 53% (65 out of 122) compared to an 86% hospitalization rate among those with osteosarcopenia (*p* < 0.001).

### 3.4. Binary Logistic Regression Analysis of Determinants of 3-Year Mortality

The binary logistic regression analysis that determined risk factors (as independent variables) for 3-year mortality (as dependent variable) is summarized in Table 4. In the first model, the analysis demonstrates that both sarcopenia and low BMD are significant predictors of increased mortality risk. Additionally, age has a notable effect with each additional year, increasing the odds of mortality by about 10%, while the female gender is also a significant predictor, with pronounced higher mortality odds in comparison to males.

In the second model, by considering osteosarcopenia as a combined condition rather than evaluating sarcopenia and low BMD separately, we observed an increased 3-year mortality rate. Specifically, osteosarcopenia increases the mortality risk by approximately 30% above sarcopenia alone and an 8% increase above low BMD, highlighting the specific relevance of osteosarcopenia as a risk factor for mortality. Other risk factors, such as age and female gender, continued to show a significant impact on mortality risk.

Further analysis incorporating nutritional status (classified as malnourished, at risk of malnutrition, and having normal nutritional status) for a subset of patients from MHH indicated a non-significant association with mortality (OR 0.700; 95%—CI 0.465–1.054; *p* = 0.088). Although not achieving statistical significance, this result suggests a trend toward better nutritional statuses being associated with a lower risk of mortality, independently from sarcopenia and osteopenia.

## 4. Discussion

This study highlights the significant impact of osteosarcopenia on 3-year mortality and hospitalization rates in older hospitalized patients, reinforcing and extending current knowledge that identifies osteosarcopenia as a critical indicator of increased vulnerability in older adults. Through binary logistic regression analysis, we have examined the effects of sarcopenia and low BMD both independently and in combination, revealing that osteosarcopenia significantly amplifies the risk of death compared to each condition on its own. Accordingly, our study suggests that the coexistence of sarcopenia and low BMD seems to be a major risk factor for mortality, underscoring the equal importance of both bone and muscle health in the overall prognosis. Although sarcopenia and low BMD increase the risk for mortality separately, their co-existence as osteosarcopenia does not multiply the risk as one might expect. This observation implies a shared underlying cause or interconnected pathophysiology between these conditions rather than completely separate risk pathways.

The interplay between bone and muscle health is closely associated throughout an individual’s lifespan. In our previous study among older hospitalized patients [25], we found that nearly every patient with sarcopenia also suffered from low BMD (90%), while conversely, only a few patients with low BMD demonstrated sarcopenia (11%), illustrating a significant asymmetrical overlap between these conditions, as has also been noted in other studies [24,32]. Despite the high prevalence of low BMD and sarcopenia among older populations, these conditions often go unrecognized and untreated [33]. As integral components of the musculoskeletal system, bone and muscle—which together constitute 55% of the body mass in a healthy adult [33,34]—engage in extensive bidirectional communication through mechanical and biochemical pathways [35,36]. This interaction is mediated by a variety of signalling molecules, including chemokines, interleukins, and growth factors, ensuring that changes in one tissue are reflected and responded to by the other [20]. With advancing age, there is a noticeable decline in both muscle and bone mass, leading to increased vulnerability to various musculoskeletal diseases.

Incorporating insights from previous studies, the importance of dietary calcium, protein, and vitamin D in the context of osteosarcopenia is profound. Calcium’s role in muscle contraction and bone health, alongside protein’s influence on muscle mass and bone density, highlights the multifaceted nature of the nutritional impact on osteosarcopenia [17,18]. Additionally, vitamin D’s mediation in muscle and bone physiology further exemplifies the intricate relationship between diet and the management of osteosarcopenia [37]. Given the controversies surrounding supplement dosage and administration, future research should aim to elucidate the optimal nutritional strategies that specifically address the needs of older adults with osteosarcopenia, integrating these dietary considerations into comprehensive management plans for osteosarcopenia.

Previous studies have established an association between osteosarcopenia and the risk of falls, frailty, hospitalization, and mortality [22,23]. In a meta-analysis integrating results from eight cohort studies involving 19,836 older individuals, osteosarcopenia was identified as a significant risk factor for fractures, falls, and mortality, with an odds ratio of 2.46 for fractures and odds ratios of 1.66 and 1.62 for mortality and falls, respectively [23]. These results are further validated by a recent comprehensive review of 66 studies, encompassing 64,404 participants, which demonstrated a pronounced impact of osteosarcopenia on similar health outcomes [38]. This latter analysis reported a pooled prevalence of 18.5% for osteosarcopenia and confirmed osteosarcopenia as a significant predictor of increased falls (HR = 1.54), fractures (HR = 2.13), and mortality (HR = 1.75) [38].

Salech and colleagues [22] conducted a study involving over 1100 individuals living in the community, with an average age of 72 years. They found that 16.4% of participants had osteosarcopenia, which was linked to a higher incidence of falls, fractures, and death. Notably, the occurrence of osteosarcopenia rose with age, affecting 33.7% of individuals aged over 80 years, and those with osteosarcopenia experienced a mortality rate of 15.9%, significantly greater than the 6.1% seen in those without this condition. Moreover, a long-term prospective study that investigated the association of sarcopenia in the presence of osteopenia with fractures and mortality over 10 years in a large sample of community-dwelling older adults showed that mortality risks were significantly higher only in participants with osteosarcopenia (RR  =  1.49, 95% CI: 1.01–2.21) compared to those without sarcopenia or osteopenia [39].

Additionally, in a recent longitudinal, population-based OPRA Cohort, which included 1044 participants who were all aged 75 at initiation and who were tracked over a decade, the use of WHO and EWGSOP2 definitions for low bone mass and sarcopenia—aligning the criteria applied in our study—unveiled significant insights [24]. The incidence of mortality was notably higher in individuals with osteosarcopenia, with 42.4% (*n* = 42) experiencing death compared to 23.9% (*n* = 117) in those with only low bone mass. Furthermore, the group identified as having osteosarcopenia showed a markedly increased 10-year mortality risk (HR 2.26 [1.46–3.51]), even after adjusting for confounders, in contrast to the low-bone-mass group, which did not exhibit a similar increase (HR 1.07 [0.75–1.55]) [24].

Reflecting the observations from the OPRA Cohort, our study likewise found that individuals with osteosarcopenia had a significantly elevated mortality rate of 46% compared to 18% among those without osteosarcopenia. Our findings demonstrate that individuals with osteosarcopenia may exhibit a higher risk of 3-year mortality when compared to those with only one of the conditions (sarcopenia or low BMD) and notably more so compared to the individuals with normal bone and muscle status. Specifically, the risk of 3-year mortality in the osteosarcopenia group was approximately 30% higher than in the sarcopenia group and 8% higher than in the low BMD group. Furthermore, when compared to individuals with normal bone and muscle status, the osteosarcopenia group showed a markedly increased risk of adverse outcomes. Our result suggests that osteosarcopenia could be considered a separate health condition, which more or less complicates sarcopenia, with its own characteristics and emphasizes the need for specific methods to diagnose it in medical settings.

While the OPRA study primarily encompassed women, our study’s demographic was similarly skewed, with female participants constituting 78% of our cohort. In our regression analysis, being female was identified as a significant predictor of mortality, showing higher odds of mortality in comparison to males. Previous research indicates a higher incidence of osteosarcopenia in women compared to men [32,38,40], attributed to hormonal changes during the post-menopause and differences in body composition that accelerate bone and muscle deterioration [41,42]. Despite this, our baseline data revealed no marked gender disparities in the rates of sarcopenia, low BMD, or osteosarcopenia [25]. This discrepancy suggests that the long-term effects of lower peak bone mass and muscle strength inherent in women, compared to men, may not be fully captured by prevalence rates alone but significantly influence mortality risk.

Altogether, the findings of this study highlight the need for the early detection and management of osteosarcopenia within clinical settings. The significant association between osteosarcopenia and increased 3-year mortality underscore the importance of integrating preventive strategies and individualized therapeutic approaches into routine geriatric care. These strategies should not only be limited to clinical and pharmacological interventions but must also consider the pivotal role of nutrition and physical activity in preventing and managing osteosarcopenia. To advance our understanding and management of osteosarcopenia, future research must prioritize longitudinal studies that integrate nutritional guidelines into the care of older adults with osteosarcopenia and explore the etiology and causes of progression and the clinical consequences of this condition among these patients. Such investigations are key to developing preventive and therapeutic strategies that will substantially improve the health outcomes and quality of life of the aging population.

Several limitations of this study should be discussed. Firstly, the 3-year follow-up period, while providing valuable initial insights, may not fully capture the longer-term outcomes associated with osteosarcopenia, a condition characterized by gradual progression and potentially delayed effects. The predominantly older female composition of our study cohort limits the generalizability of our results across the broader spectrum of the older adult population, particularly men. The methodology of utilizing telephone interviews for follow-up data collection introduces the potential for recall bias, as patients or their relatives may not accurately recall health events and conditions. Additionally, the presence of unaccounted confounding variables, such as socioeconomic factors, cognitive function, disease severity, and lifestyle behaviours, could influence the observed associations between osteosarcopenia and health outcomes. Another limitation is the lack of detailed dietary analyses in our study design. The role of nutrition in the progression and management of osteosarcopenia is undeniable, with dietary factors such as protein intake, calcium, and vitamin D intake being crucial for muscle and bone health. Our study’s focus on clinical and epidemiological aspects, without a comprehensive evaluation of nutritional factors, underscores the necessity for future research to include these elements. Such integration would enable a more comprehensive understanding of osteosarcopenia and potentially guide more effective prevention and treatment strategies. Moreover, given that our study was focused on older hospitalized patients who are more likely to exhibit higher rates of multimorbidity than the general older adult population, the applicability of our findings to those with different health profiles may be limited. This focus underscores the need for broader studies that encompass a wider array of settings and populations to enhance the understanding and management of osteosarcopenia in diverse older adult groups.

## 5. Conclusions

This study demonstrates that osteosarcopenia is a frequent condition among older hospitalized patients and that it is associated with an increased 3-year mortality risk compared to either sarcopenia or osteoporosis alone. The prevention, early comprehensive evaluation, and treatment of both general bone and muscle disorders, alongside nutritional optimization, are crucial to counteract the inherent risk of both conditions.

## Figures and Tables

**Table 1:** Characteristic of the study population.

	Baseline Data (*n* = 572)
Gender (*n*, %)	
Female	449 (78)
Male	123 (22)
Age (year)	75.1 ± 10.8
Height (m)	1.64 ± 0.08
Actual body weight (kg)	74.0 ± 15.6
BMI (kg/m^2^)	27.3 ± 5.3
Geriatric assessment	
^a^ MNA-SF, median (IQR)	9 (7–11)
Malnourished (*n*; %)	72 (26)
At risk of malnutrition (*n*; %)	141 (52)
Normal nutritional status (*n*; %)	60 (22)
Handgrip strength (kg)	22.6 ± 10.8
Frail scale, median (IQR)	2 (1–3)
SARC-F scores, median (IQR)	4 (2–6)
SPPB, median (IQR)	9 (5–10)
AMM (kg)	18.4 ± 4.1
Bone mass density (T-score)	−1.8 ± 1.3

^a^ MNA-SF, Mini Nutritional Assessment Short, which was measured in 273 patients from MHH; SPPB, short physical performance battery; AMM, appendicular muscle mass (muscle mass of the arms + muscle mass of the legs). Frail simple, SARC-F and SPPB were measured in 558, 561, and 302 subjects. Values are given as mean ± SD, median (interquartile range), or number (%).

**Table 2:** Post-discharge patient outcomes, including mortality and hospitalization events.

	Post Discharge Outcomes
	*n * = 533
^a^ Overall mortality during follow-up	159 (30)
3-year mortality	106 (20)
	*n * = 490
Overall unplanned hospitalization	295 (60)
Overall unplanned hospitalization due to fall	70 (14)
Overall unplanned hospitalization due to fracture	41 (8)

^a^ The median follow-up duration for the study was 4 years, with an interquartile range of 4 to 5 years, and the minimum follow-up period was 3 years.

**Table 3:** Comparison of outcomes data between groups.

	Sarcopenia	No Sarcopenia	*p* Value
	Total (*n* = 46)	Total (*n* = 487)	
3-year mortality (*n* = 533)	19 (41)	87 (18)	<0.001
	Total (*n* = 41)	Total (*n* = 449)	
Unplanned hospitalization (*n* = 490)	34 (83)	261 (58)	0.001
Unplanned hospitalization due to fall (*n* = 490)	8 (19)	62 (14)	0.349
Unplanned hospitalization due to fracture (*n* = 490)	6 (15)	35 (8)	0.139
	Low BMD	Normal BMD	*p* value
	Total (*n* = 398)	Total (*n* = 135)	
3-year mortality (*n* = 533)	94 (24)	12 (9)	<0.001
	Total (*n* = 363)	Total (*n* = 127)	
Unplanned hospitalization (*n* = 490)	227 (62)	68 (53)	0.047
Unplanned hospitalization due to fall (*n* = 490)	63 (17)	7 (5)	<0.001
Unplanned hospitalization due to fracture (*n* = 490)	35 (10)	6 (5)	0.057
	Osteosarcopenia	No Osteosarcopenia	*p* value
	Total (*n* = 41)	(*n* = 492)	
3-year mortality (*n* = 533)	19 (46)	87 (18)	<0.001
	Total (*n* = 36)	Total (*n* = 454)	
Unplanned hospitalization (*n* = 490)	31 (86)	264 (58)	<0.001
Unplanned hospitalization due to fall (*n* = 490)	8 (22)	62 (14)	0.211
Unplanned hospitalization due to fracture (*n* = 490)	6 (17)	35 (8)	0.069

BMD; bone mineral density. Values are given as numbers (%).

**Table 4:** Binary regression analysis of risk factors associated with 3-year mortality.

	3-Year Mortality					
				95% CI for Exp(B)		
Model 1	B	Std. Error	Exp(B)	Lower	Upper	*p* Value
Sarcopenia (yes/no)	0.759	0.377	2.135	1.020	4.472	0.044
Low BMD (yes/no)	0.946	0.362	2.576	1.267	5.235	0.009
Gender (female/male)	0.842	0.273	2.322	1.359	3.967	0.002
Age (year)	0.098	0.016	1.103	1.069	1.139	0.001
BMI (kg/m^2^)	0.011	0.024	1.011	0.964	1.061	0.651
**Model 2**						
Osteosarcopenia (yes/no)	1.022	0.392	2.778	1.291	5.992	0.009
Gender (female/male)	0.776	0.269	2.173	1.283	3.680	0.004
Age (year)	0.102	0.016	1.108	1.074	1.143	0.001
BMI (kg/m^2^)	0.004	0.024	1.004	0.957	1.054	0.856

BMD; bone mineral density.

## Data Availability

The datasets analyzed during the current study are available from the corresponding author upon reasonable request due to legal reasons.
